# A commentary on the practice of using the so-called typeless species

**DOI:** 10.3897/zookeys.693.10945

**Published:** 2017-08-23

**Authors:** Anatoly I. Shatalkin, Tatiana V. Galinskaya

**Affiliations:** 1 Zoological Museum, Lomonosov Moscow State University, 6 Bol’shaya Nikitskaya St., Moscow, 125009, Russia; 2 Lomonosov Moscow State University, Biological faculty, Entomology department, Leninskie gory 1-12, Moscow, 119234, Russia

**Keywords:** ICZN, *nomen nudum*, nomenclatural type, nomenclature, typeless and standard species

## Abstract

The fears expressed by Santos et al. (2016) that description of typeless species (new species described based on field photographs) can be fatal for the practice of taxonomy which will succumb to an uncontrollable stream of “species of questionable delimitation” are, in our opinion, exaggerated. The Code already protects taxonomic practice from subjectivity quite well by limiting opportunities for descriptions of new species based on field photos by rigid requirements, and only skilled taxonomists with extensive knowledge of a group are capable of fulfilling them. If a taxonomist has omitted to compare the new typeless species with the known species externally similar to it, the latter cannot be diagnosed and its name in that case becomes *nomen nudum*. Typeless species can coincide with species described earlier, but can represent a new species differing in internal features. To describe typeless species without infringement of Article 13.1 a taxonomist should compare this species to all related and similar species described earlier.

## Introduction

In 2015, Marshall and Evenhuis published a description of a new African species *Marleyimyia xylocopae* (Diptera, Bombyliidae), which was based on a photograph taken in the field. The following year another article was published with a description of a new species *Nothybus absens* (Diptera, Nothybidae) based on two photographs taken in China (Lonsdale and Marshall 2016). This species also does not have a preserved holotype. To designate a species described without a reference to type specimens deposited in the collection, we will use the term ‘typeless species’ (Santos et al. 2016).

Marshall and Evenhuis (2015, p. 119) have clearly specified the two conditions that should be followed when describing a species on the basis of photographs alone, without a preserved specimen as a holotype: “even in the absence of a collected type specimen, current technologies such as high-resolution photography can often provide enough information for a proper description resulting in a readily recognizable and unequivocally distinct newly named species”. Therefore, a photograph should (1) provide enough information (2) to differentiate unequivocally new species from other already described species. However, a photograph can turn out to be insufficient when internal structures and other morphological features not shown in the photograph are used for species discrimination (Cianferoni and Bartolozzi 2016, p. 129). In such cases it simply cannot be used for the description of species.

It is important to emphasize one more detail. Aside from its valuable function as the bearer of a taxon name (Article 61.1: “Each nominal taxon in the family, genus or species groups has actually or potentially a name-bearing type”), the type also plays an equally important role of being the carrier of objective data about a species. This function allows one to reevaluate the adequacy and accuracy of initial description of a species by a repeated study of its holotype. Consequently, the need for this function can be partly dismissed if a series of high quality photographs are used since they convey the information about a species more accurately in comparison to verbal and written descriptions.

According to the second condition, a taxonomist should discriminate the “new insect species described and named solely on the basis of field photographs of the type specimen” from all the previously described known species (Marshall and Evenhuis 2015, p. 119); in this case the type of the new species is a specimen illustrated in the photograph (article 73.1.4). Surprisingly, the Code specifically demands a fulfillment of this condition for description of species by photograph, and this will be shown later. As for the claimed impossibility of strict discrimination of a species in case of finding a sibling species in the future (Cianferoni and Bartolozzi 2016), this problem is not new to systematics. As Marshall and Evenhuis (2016, p. 88) have emphasized, “every time we examine a previously named species in the course of a revision there is a possibility that it will turn out to be two or more species, and that the additional species will be undescribed. A normal part of that process is to figure out which of the multiple species corresponds with the original name”. In case of sibling species, we cannot unequivocally establish which of two species corresponds with the original name. This case is also not a serious issue for a taxonomist. For example, a putatively new insect species has been discovered, which, when relying solely on the description, does not differ from another species described earlier, but nothing but a pin has remained from a type specimen of the latter. In this case the entomologist must formally connect one of the two species with an earlier described species at his own discretion.

At the same time we cannot completely agree with another statement of the authors (Marshall and Evenhuis 2015, p. 120), that “We expect that such descriptions [‘of species without preserved type specimens’] that do not render new species unequivocally recognizable will be rejected, just as they should be if they were based on dead type specimens”. The first part of the statement is supported by the Code, as we will show later. As to the second part of sentence, the Code does not impose any restrictions on the traditional practice of the description of species. In any case, in the differential diagnosis does not demand full discrimination of a new species from all the previously described species.

Among entomologists, the reaction to the practice of describing new species based on a photograph only, without the specimens deposited in a collection, was both positive (Pape 2016) and negative (Santos et al. 2016).

## Critical comments on the practice of description of typeless species

The publication of Marshall and Evenhuis (2015) has been critically analyzed by a collective of authors (Santos et al. 2016, p. 513), who have concluded that “the idea of associating names with beautiful species of questionable delimitation based only on photographs may be highly damaging to the practice of taxonomy” that “adjustments and corrections to the ICZN (Anonymous 1999; further referred to as the Code, or ICZN) especially to Article 73.1.4, are necessary and urgent. A modification to the ICZN would prevent the creation of other species names based solely on illustrations or photographs without real and proper type specimens”. Amorim et al. (2016, p. 125) have also expressed disagreement with the practice of description of species without having a preserved holotype: “The spirit of the International Code of Zoological Nomenclature is willfully violated by a description based only on a photograph”. In this work authors analyzed the answers given by Marshall and Evenhuis (2015) to critical remarks addressed to them. In their critical analysis Amorim et al. (2016, p. 123) moved from the two following positions: “1. A specimen, as a standard of its species, has infinitely more fidelity than images thereof…”, and “2. Subsequent examination of an image is unlikely to find any new characters beyond what the pixels that were originally captured have already revealed”.

We completely agree with these statements. However, at the same time these fix the particular nomenclatural status of typeless species (the term offered by Santos et al. 2016), which is connected with serious restrictions in a choice of traits to be described. These restrictions have a double nature. Firstly, these species can be described by a finite set of mainly external characteristics. This means that from the taxonomic standpoint the information provided by a photograph can be insufficient for promoting a hypothesis about a new species. If, for example, some species in a group differ in the structure of genitalia, then a photograph will be insufficient to make a judgement about the species shown in it. This is the simplest case. Secondly, there are restrictions imposed by the Code. Upon instigating the description of a species by a photograph, a taxonomist should understand whether he infringes any articles of the Code. In other words, he should solve a following question: what requirements are made by the Code for the practice of the description of species by photographs? This question will be central in our analysis.

Löbl et al. (2016, p. 84) have expressed misgivings concerning the practice of description of species by a photo believing that “Taxonomy might be threatened because of the increasing power and availability of digital photography, improving one’s chances of quickly capturing images of animals, without carefully studying the animal themselves”. This conclusion has something in common with the opinion of Cianferoni and Bartolozzi (2016, p. 129): “These new practices could make it much easier to describe new species, and this in turn would contribute to the increasing involvement of unsuitably qualified people. A number of inexperienced naturalists or even nature photographers with no biological training could begin to submit descriptions to journals, based only on photographs”.

Entomologists were not the first who ventured to describe new species without type specimens deposited in a collection before scientific community. Earlier three works with such descriptions of new species of primates have been published: the mangabey *Lophocebus kipunji* (Jones et al. 2005), the lemur *Avahi cleesei* (Thalmann and Geissmann 2005); and the capuchin *Cebus queirozi* (Mendes Pontes et al. 2006). These works also were a subject of active discussion. Its results have been summarized by Dubois and Nemésio (2007): “Whereas some authors (Timm et al. 2005, Landry 2005, Oliveira and Langguth 2006) stated that the *nomina* of these taxa [the new species of primates mentioned above and some others], if published after 1999, are nomenclaturally unavailable, others (Wakeham-Dawson et al. 2002, Polaszek et al. 2005) defended the opposite idea”.

Timm et al. (2005, p. 2163) wrote “The photographs are not valid substitutes for a type specimen. The function of a type specimen in nomenclature is to provide an objective basis for the application of a species-group name”. While agreeing with these thoughts, it is nonetheless necessary to note that this objective basis becomes actual only in the presence of material for comparison. However, what if this material is absent? Or if a species is very rare? If someone had the luck to photograph it, we cannot wait for a next happy opportunity to see it. Here is a very symptomatic example that testifies to the rarity of some dipterous species. The flat-footed fly *Agathomyia aurantiaca* (Bezzi, 1893) (Diptera, Platypezidae) was described on a sole male from Monte Baldo (Northern Italy), caught 19. IX.1891 on a leaf of coltsfoot *Tussilago farfara* L., 1753. While this record of this species is still the only one for Europe (Chandler 2001, p. 135), our colleague Michal Tkoč (Natural History Museum, Praha) kindly informed us that he found one specimen of this species caught at the beginning of the last century and stored in MNHN (Paris). The only small series of males and females of this species had been collected in the vicinities of the town of Zeya (the Amur area). Male *A. aurantiaca* possess a unique coloring, and can be easily differentiated from all the other species based on a photograph.

Of course, it is possible to take a different point of view and claim that there is a shortcoming in the Code, that the mentioned authors had found loopholes in the Code, and that the problem will be resolved if these loopholes are accounted for. We are, however, inclined to take a compromising position. Being the practical document regulating the work of systematists, in a number of cases the Code is forced to go for conciliatory proposals that consider the interests of different sides. The history of description of the highland mangabey *Lophocebus kipunji* (Jones et al. 2005) from southern Tanzania based on two photos shows this well (Polaszek et al. 2005). We completely agree with Polaszek et al. (2005, p. 2166) who wrote “The allowance under the Code for designation of living specimens as holotypes needs to be more widely recognized, given contemporary concerns for the conservation of threatened species. There is no doubt that many newly described taxa will be threatened (*L. kipunji* will be designated as “critically endangered” in the IUCN Red List). Dead animal specimens should not be understood to be essential to the process of establishing new taxa. In such cases, supplementation with evidence such as sonograms and oscillograms of species-specific vocalizations, and molecular information (now readily derived from noninvasive samples, e.g., hair, urine, and feces) may contribute to validation.”

Some conceptual ambiguity is certainly present in the Code. On the one hand, type specimens should be deposited in a collection, yet on the other hand this norm is not always obligatory. However, there is an understanding that it is caused by serious reasons, first of all related to the responsibility of the world community that has set for itself the task of preserving the fauna of our planet: “Due to the declining abundance of many species, access to complete anatomical specimens is becoming a vanishing luxury. (...) For many animal taxa, the lethal collection of such voucher specimens would now also be considered unethical” (Dalebout et al. 2004, p. 459). At the same time there is a danger to taxonomy in that these special cases can become the rule, and in hands of unskilled taxonomists will lead to nomenclatural chaos. That is exactly what the Code should prevent.

## Typeless species and Article 13.1

Now, the basic question stated in works of Santos et al. (2016) and by other zoologists touches upon possible damage for taxonomy from the practice of describing of new species based on photographs alone. In our opinion, the expressed apprehensions that many zoologists, and not only these, will instantly go for the opportunity to describe species based on field photographs, and as the result taxonomy will become flooded with typeless species, are a little exaggerated.

In our opinion, Article 13.1 of the Code imposes serious restrictions on the practice of describing of new species based on photographs. It establishes the following requirements with regard to the availability of names for new taxa:

• 13.1. Requirements. To be available, every new name published after 1930 must satisfy the provisions of Article 11 and must

• 13.1.1. be accompanied by a description or definition that states in words characters that are purported to differentiate the taxon…

Accordingly, in some insect groups new species of insects clearly cannot be described with only photographs. Species that are distinguished by structure of genitalia are an obvious example. One may object that, after all, Article 13.1.1 does not mandate that we use genitalia for differentiation of species. For further clarity we provide the following hypothetical example.

Let us consider two species of flies, *A* and *B*, belonging to some genus. Both have been described on the basis of external characters. Let us assume further, that one more species *C* has been found and this species differs from species *B* externally, and from species *A* only in a structure of genitalia. Article 13.1.1 of the Code does not limit taxonomists in the number of characters deemed to be necessary and sufficient for strict differentiation of a new species. That means that if a taxonomist had missed one or several known species in his differential diagnosis for a new species, the Code does not see any nomenclatural infringements. However, if among these missed species there was a species (for example species *A*) which does not differ from a new species *C* in the characteristics used, the consequences will be different for the practice of description of a species based on photograph, vs. the traditional practice of description of a species with a fixed specimen as a holotype. In the second case we simply repeatedly examine holotypes of new (*C*) and known (*A*) species, analyzing all accessible characteristics, and including new ones that were not used earlier for the differentiation of the species. In any case we will receive a certain result: either the species do not differ (in this case the name of a new species becomes a synonym of the known one), or their independent status is confirmed.

In a similar situation, in the case of a species without extant specimens as a holotype we will not receive an unequivocal result. If a new typeless species *C*, about the characteristics of which we judge only by photographs, does not differ from the species *A*, it does not confirm the identity of these species. They may be distinguished by other characters that are invisible in a photograph; for example, by details of genitalia. However, it also does not mean that these species are distinguished, even if the holotype of a new species *C*, i.e., that single specimen portrayed on a photograph, really differs from a known species *A* in the structure of genitalia. It is impossible to solve this issue by having only a photograph of the holotype *C*. Hence it follows that a taxonomist describing a new species by a photograph did not differentiate it, and now it is impossible to correct his mistake. As the result of nonprofessional actions of the taxonomist, the species described by him on the grounds of a photograph cannot be differentiated in principle, i.e. it cannot be conclusively attributed neither to a new species *C*, nor to the known species *A*. By virtue of this the name of a new species, since it is impossible to diagnose this species, is, under the Code, a ‘*nomen nudum*’.

According to the Code Glossary, ‘*nomen nudum*’ is “a Latin term referring to a name that, if published before 1931, fails to conform to Article 12; or, if published after 1930, fails to conform to Article 13”. In our example of typeless species *C*, that is exactly what takes place: the zoologist did not differentiate species *C*, and thereby has not executed the requirement of Article 13.1.

So, when describing a new typeless species a specialist should differentiate it from all the species described earlier. If the taxonomist in the differential diagnosis misses the comparison to a known species with which a typeless species is similar externally, the latter cannot be diagnosed on any account, and therefore its name falls under the definition of *nomen nudum*. Once again, we need to emphasize that *nomen nudum* here would be the result of nonprofessional actions of the taxonomist, who should have formally approached the task of comparison of typeless species to the closely related species described earlier.

Thus, conditions of the description of a typeless species are more rigid, and can be granted only by a professionally established specialist with good knowledge of the studied group. Let us remember that for the traditional procedure of a species description with deposition of fixed reference specimens in public collections the Code does not demand a comparison of a new species to all the species described earlier and belonging to the same genus or closely related genera. What are the causes of these serious differences in the practice of new species description?

The particularity of taxonomic names introduced based on photographs is that they have a substantial double interpretation connected in the example given above, solved by explaining formulae: “species *C*” and “typeless species *C*”, where *C* designates the photographed specimen; for example, the holotype of *Marleyimyia xylocopae*. The first name designates taxon *C* as it is, in its all completeness and with all attributes inherent to it. That is to say that the name *Marleyimyia xylocopae* is considered without any connotation. The second name designates taxon C connotatively, giving its narrow definition by the means of a finite set of characters that can be recognized in a photograph. According to the accepted assumption, taxon *C* does not differ from taxon *A* in these characteristics. Hence it follows that the name “typeless species *C*” (in other words connotation of the name *Marleyimyia xylocopae*) can simultaneously designate both the taxon *C* and the taxon *A*. Once again, we shall emphasize that it will become possible only in a case when a taxonomist describing a typeless species had omitted to compare it to a similar species described earlier. This hypothetical opportunity has no relation to the real species *Marleyimyia xylocopae* (described by Marshall and Evenhuis 2015), as authors followed the requirements of Article 13.1. There is no such species indistinguishable from *Marleyimyia xylocopae* as the species *A* described above. The name *Marleyimyia xylocopae* is lawful from the point of view of the Code.

How, according to Article 13.1, is the problem of differentiation of taxa solved in cases of standard species based on extant specimen as holotype? Let us assume that a taxonomist describing a new species *D* had not compared it to the known species *E*, which is similar to first one in external attributes. It is easy to see that the mistake of this taxonomist can always be corrected, and consequently it does not make the taxonomic position of species *D* uncertain, as it can be in cases of typeless species. Even though the species *D* and *E* do not differ in external characteristics, they can be distinguished by other attributes; for example, their genitalia or the characters of their genome. A taxonomist can repeatedly examine the holotypes of these species to solve the problem of taxonomic status for the species *D*. The latter can either appear identical to the species *E*, or will represent a separate species. The name “species *D*” does not bear any limiting connotation; therefore, taxon *D* is diagnosable in our example.

Due to the constantly extending character database of systematics, the inclusion of new categories of features in its arsenal, and additionally due to the continuous activity of taxonomists describing new species, the Code fundamentally cannot formalize Article 13.1.1. Therefore, it cannot establish the number of characteristics or species that would be necessary and sufficient for differentiation of a new species from related or similar ones. Consequently, the application of Article 13.1.1 is completely left at the discretion of taxonomists (see Recommendation 13A), and it will depend only on his readiness to seriously approach the compiling of a differential diagnosis, as well as on his desire to analyze all the currently available information on closely related species.

The seeming impression that nomenclature is not connected to taxonomy comes from this general impossibility of formalizing the procedure of description of new species. Actually, it is incorrect, there is a connection; however, it is mediated in many cases. At the same time, the analysis of names of typeless species shows that this connection can be clearly expressed.

## Taxonomy and nomenclature

Dubois and Nemésio (2007) have expressed the opinion that “the definition of taxa is not a matter of nomenclature but a matter of taxonomy, and the Code does not deal with taxonomy but with nomenclature”. It is possible to agree with the first part of the sentence. As for the second part of that sentence, the statement by Dubois and Nemésio (2007) does not, in our opinion, take into account the full extent of the primary principle of the Code: “The Code refrains from infringing upon taxonomic judgement, which must not be made subject to regulation or restraint.” We claim that the Code is a practical guide for the description of new taxa. The connection between nomenclature and taxonomy is created through this practice.

Nomenclature is often defined as the practice of designation of taxa. This definition considers taxonomy and nomenclature to be two different and disconnected aspects of the work of systematists. It is assumed that a systematist had discovered a new taxon, i.e., he found that it differs from all other taxa known at that time, and consequently provided a name for the new taxon. It is difficult to agree with such an interpretation. Here we follow the opinion of I. Ya. Pavlinov, who wrote in his manual of taxonomic nomenclature (2015, p. 11): “Despite the traditional separation of two “foundations” of taxonomy recognized by Linnaeus, viz. classification (disposition) and naming, the nomenclatorial codes actually regulate both. The reason is quite simple and obvious: as there cannot be taxa appearing in scientific classifications and texts without being properly nominated, the “empty” *nomina* that are not properly assigned to particular taxa do not have any biological meaning.”

The Code of Nomenclature was written by taxonomists for taxonomists studying various groups of organisms, aiming to order and by that to facilitate, as much as possible, their activity with regard to the descriptions of taxa. The procedure for descriptions of new taxa is regulated in a number of places within the Code, and in particular the terms of availability of a taxonomic name are stipulated in Article 13.1. All things considered, the nomenclature in systematics is a system of rules created for formal descriptions and designations of new taxa. Formal requirements for the procedure of describing new taxa are aimed at avoiding the mistakes of naming, i.e., homonyms, synonyms, and *nomina nuda* (Principles 3, 5; Glossary).

As it has already been stated, Article 13.1.1 cannot be formalized with relation to the practice of description of standard species. With all this, the Code brings attention that he might act negligently within the requirements of this article, and the result of his oversight could be a synonymy between the name of a new species newly established and a species described earlier. In reference to typeless species described by photographed individuals, the Code implies severe constraints concerning the procedure of comparison of new species to related or similar species. In case of noncompliance with these conditions *nomina nuda* (see above) as well as synonyms can be created.

To date there are almost no examples of a *nomen nudum* created in consequence of the description of a typeless species. One of such example was the description of *Nembrotha yonowae* Goethel & Debelius, 1992, based on photo; later Nathalie Yonow (1994) made mention that this species is *nomen nudum*; subsequently, Pola et al. (2008) picked up on it and reinstated the name *Nembrotha yonowae*, while describing more species in the complex. Hence, the status of this species is still under consideration. An example of synonymy is the name *Strix omanensis* recently established for a new species of desert owl from northern Oman (Robb et al. 2013). Authors based the description of *Strix omanensis* on photos; as Marshall and Evenhuis (2016, p. 88) have emphasized “Robb et al. recognized that there were two species under the name *Strix butleri* (Hume), and that one of them must therefore be new. Unfortunately, they misidentified the new species as the one corresponding to the original name even though there was a type specimen corresponding to that 1878 name, and this led them to coin a new name for *S. butleri*”.
